# Plasma and Urine Free Glycosaminoglycans as Monitoring Biomarkers in Nonmetastatic Renal Cell Carcinoma—A Prospective Cohort Study

**DOI:** 10.1016/j.euros.2022.06.003

**Published:** 2022-06-29

**Authors:** Francesco Gatto, Saeed Dabestani, Sinisa Bratulic, Angelo Limeta, Francesca Maccari, Fabio Galeotti, Nicola Volpi, Ulrika Stierner, Jens Nielsen, Sven Lundstam

**Affiliations:** a Department of Biology and Biological Engineering, Chalmers University of Technology, Gothenburg, Sweden; b Department of Translational Medicine, Division of Urological Cancers, Lund University, Kristianstad Central Hospital, Region Skane, Lund, Sweden; c Department of Urology, Kristianstad Central Hospital, Region Skane, Kristianstad, Sweden; d Department of Life Sciences, University of Modena and Reggio Emilia, Modena, Italy; e Department of Oncology, Sahlgrenska University Hospital, Gothenburg, Sweden; f BioInnovation Institute, Copenhagen N, Denmark; g Department of Urology, Sahlgrenska University Hospital, Gothenburg, Sweden

**Keywords:** Glycosaminoglycans, Liquid biopsy, Renal cell carcinoma, Tumor biomarkers

## Abstract

**Background:**

No liquid biomarkers are approved in renal cell carcinoma (RCC), making early detection of recurrence in surgically treated nonmetastatic (M0) patients dependent on radiological imaging. Urine- and plasma free glycosaminoglycan profiles—or free GAGomes—are promising biomarkers reflective of RCC metabolism.

**Objective:**

To explore whether free GAGomes could detect M0 RCC recurrence noninvasively.

**Design, setting, and participants:**

Between June 2016 and February 2021, we enrolled a prospective consecutive series of patients elected for (1) partial or radical nephrectomy for clinical M0 RCC (cohort 1) or (2) first-line therapy following RCC metachronous metastatic recurrence (cohort 2) at Sahlgrenska University Hospital, Gothenburg, Sweden. The study population included M0 RCC patients with recurrent disease (RD) versus no evidence of disease (NED) in at least one follow-up visit. Plasma and urine free GAGomes—consisting of 40 chondroitin sulfate (CS), heparan sulfate, and hyaluronic acid (HA) features—were measured in a blinded central laboratory preoperatively and at each postoperative follow-up visit until recurrence or end of follow-up in cohort 1, or before treatment start in cohort 2.

**Outcome measurements and statistical analysis:**

We used Bayesian logistic regression to correlate GAGome features with RD versus NED and with various histopathological variables. We developed three recurrence scores (plasma, urine, and combined) proportional to the predicted probability of RD. We internally validated the area under the curve (AUC) using bootstrap resampling. We performed a decision curve analysis to select a cutoff and report the corresponding net benefit, sensitivity, and specificity of each score. We used univariable analyses to correlate each preoperative score with recurrence-free survival (RFS).

**Results and limitations:**

Of 127 enrolled patients in total, 62 M0 RCC patients were in the study population (median age: 63 year, 35% female, and 82% clear cell). The median follow-up time was 3 months, totaling 72 postoperative visits —17 RD and 55 NED cases. RD was compatible with alterations in 14 (52%) of the detectable GAGome features, mostly free CS. Eleven (79%) of these correlated with at least one histopathological variable. We developed a plasma, a urine, and a combined free CS RCC recurrence score to diagnose RD versus NED with AUCs 0.91, 0.93, and 0.94, respectively. At a cutoff equivalent to ≥30% predicted probability of RD, the sensitivity and specificity were, respectively, 69% and 84% in plasma, 81% and 80% in urine, and 80% and 82% when combined, and the net benefit was equivalent to finding an extra ten, 13, and 12 cases of RD per hundred patients without any unnecessary imaging for plasma, urine, and combined, respectively. The combined score was prognostic of RFS in univariable analysis (hazard ratio = 1.90, *p* = 0.02). Limitations include a lack of external validation.

**Conclusions:**

Free CS scores detected postsurgical recurrence noninvasively in M0 RCC with substantial net benefit. External validity is required before wider clinical implementation.

**Patient summary:**

In this study, we examined a new noninvasive blood and urine test to detect whether renal cell carcinoma recurred after surgery.

## Take Home Message

No liquid biomarkers are approved in renal cell carcinoma (RCC), making early detection of recurrence events following surgery of nonmetastatic RCC difficult. Here we develop a noninvasive blood/urine test measuring free glycosaminoglycans that can detect RCC recurrence events.

## 1 Introduction

Renal cell carcinoma (RCC) is the most common kidney malignancy and accounts for about 174 000 deaths annually worldwide [1]. Nonmetastatic RCC (M0 RCC) is largely curable by surgical resection via partial or radical nephrectomy, the recommended treatment for the majority of patients [2]. However, 20–30% of all M0 RCC cases experience postsurgical recurrence within 5 year [3]. Current follow-up strategies aim at early detection of recurrence because symptomatic recurrences have poorer prognosis than recurrences detected within a follow-up protocol [4]. Despite the importance of follow-up, there is disagreement in the selection of the optimal strategy. Current protocols largely depend on radiological imaging, typically chest/abdomen computed tomography every 6–12 months up to 3–5 year depending on the recurrence risk after surgery [5]. There is evidence that imaging is currently offered too intensely with little survival benefit [6]. Conversely, long-term recurrences after 5 year from initial surgery occur in a non-negligible fraction of patients [7].

Noninvasive detection of recurrence using molecular biomarkers could radically improve follow-up. However, currently neither molecular nor liquid biomarkers are approved in the clinical management of RCC [8], [9]. Liquid biopsies are an emerging class of cancer diagnostics based on biofluidic tumor-related biomarkers, for example, circulating free DNA (cfDNA) [9]. However, cfDNA has so far shown disappointing yields in RCC [10]. Recent developments using methylated cfDNA and metabolomics are promising [11], [12], but these assays are complex and the availability of standardized kits remains years away.

Glycosaminoglycans (GAGs) are structurally diverse polysaccharides. Their complex profile of sulfation and epimerization patterns—also called the GAGome—is now viewed as a nongenetic code that controls a wide range of biological functions, with many of these being active in cancer [13], [14]. We and others observed that genetic alterations specific to RCC correlated with significant reprogramming of cell metabolism [15], [16], [17], [18]. Using a systems biology approach, we identified GAG biosynthesis as the most deregulated metabolic pathway in RCC [19]. In exploratory prospective and retrospective case-control studies, plasma and urine GAGomes were significantly altered in RCC patients compared with healthy individuals [15], [16], [17]. Successively, we verified the analytical performance of an ultra-high-performance liquid chromatography and triple-quadrupole mass spectrometry (UHPLC-MS/MS) kit for GAGome analysis, which selectively measures the protein-free fraction or free GAGomes [18]. As opposed to total GAGomes in previous studies [15], [16], free GAGomes had stable and robust reference levels in healthy adults [19].

In this study, we sought to explore whether urine and plasma free GAGomes correlated with postsurgical recurrence in M0 RCC in a single-center prospective cohort study.

## 2 Patients and methods

### 2.1 Study design

This study is reported in compliance with the STARD guidelines (Supplementary material). The present study was approved by the Ethical Committee (Etikprövningsmyndigheten) in Gothenburg, Sweden, in February 2016 (047-16). The study was registered in clinicaltrial.gov (identifier: NCT02732652). The study followed a double-blind single-center prospective cohort design. Two cohorts were defined: (1) patients elected for partial or radical nephrectomy for suspected RCC (cohort 1) and (2) patients elected for first-line therapy following metachronous metastatic RCC recurrence (cohort 2; see the eligibility criteria in the Supplementary material). Participants were enrolled at the Sahlgrenska University Hospital, Gothenburg, Sweden, between June 2016 and February 2021. Eligible participants were identified based on a referral to partial or radical nephrectomy for suspected RCC in cohort 1, and on a referral to first-line systemic therapy for metastatic RCC in cohort 2. Both cohorts formed consecutive series. In cohort 1, after surgery, patients were followed up with radiological imaging twice a year for 2 year and then annually for up to 5 year as per the institutional guidelines. The patients provided EDTA-plasma and urine samples for GAGome analyses before surgery and at each postsurgical follow-up visit in cohort 1, and before treatment start in cohort 2. Radiological imaging was performed using chest and abdomen plain and contrast-enhanced computed tomography or magnetic resonance imaging in patients with contrast medium allergies or renal insufficiency. Recurrence status was assessed by the investigator (S.L.) as recurrent disease (RD) or no evidence of disease (NED). In cohort 2, all patients were classified to have RD. Further details on the assessment of recurrence status are described in the Supplementary material.

### 2.2 Free GAGome measurements

Whole blood samples were collected in EDTA-coated tubes at room temperature and centrifuged within 15 min (2500*g* for 15 min at 4°C). Urine was collected in polypropylene tubes at room temperature. All samples were stored at –70°C and shipped on dry ice. The free GAGome analysis was performed at a single blinded central laboratory (Lablytica Life Sciences AB, Sweden) using the MIRAM® Free Glycosaminoglycan Kit (Elypta AB, Sweden), wherein the absolute concentrations (in µg/ml) of 17 chondroitin sulfate (CS), heparan sulfate, and hyaluronic acid disaccharides—that is, the free GAGome features—were detected and quantified using a UHPLC-MS/MS system (Acquity I-class Plus Xevo TQ-S micro, Waters™, United States) following the kit instructions for use (Supplementary material) [18]. Outlier samples were identified and excluded. Nondetectable disaccharides (<0.1 µg/ml on median) were not considered as features in downstream analyses.

### 2.3 Correlation analyses

Each detectable feature in the plasma and urine free GAGome was correlated with recurrence status (RD vs NED), as assessed at the same postoperative visit in which the corresponding plasma or urine sample was collected using a Bayesian mixed-effect logistic regression model (Supplementary material). Each preoperative GAGome feature, as measured in all patients elected for partial or radical nephrectomy for RCC from cohort 1, correlated with the following histopathological variables using univariable logistic or ordinal Bayesian regression (Supplementary material): tumor size, Fuhrman nuclear grade, tumor-node-metastasis (TNM) stage, malignant or benign lesion, and RCC subtype.

### 2.4 Development of free CS RCC recurrence scores

The top two free GAGome features were used as inputs to train two Bayesian Additive Regression Trees (BART) models [20]—one for plasma and the other for urine. A third BART model was developed by using as inputs the outputs of the plasma and urine BART models (Supplementary material). The output from each BART model is equivalent to the predicted probability of RD (in %), and it was named plasma, urine, or combined free CS RCC recurrence score.

### 2.5 Statistical analysis for the diagnostic performance of free CS RCC recurrence scores

We performed internal validation using bootstrap resampling and reported the predictive performance in terms of scaled Brier score (%) and Nagelkerke *R*^2^ (%) with empirical 95% confidence intervals (CoIs). We assessed the discrimination performance in terms of the area under the receiving operating characteristic curve (AUC) for the classification of RD versus NED according to each score versus the observed recurrence status. The goodness of fit and corresponding χ^2^ statistics were tested using the Hosmer-Lemeshow test. We used a decision curve analysis to determine a single test positivity cutoff for all free CS RCC recurrence scores, so that the net benefit over the base scenarios “intervention for all” (ie, radiological evaluation for all) and “intervention for none” (ie, radiological evaluation for none) was maximized [21]. We determined the clinical usefulness of each score by cross-tabulating RD versus NED as predicted by the free CS RCC recurrence score versus the recurrence status. We performed three distinct analyses, one per score (plasma, urine, and combined), to determine its sensitivity and specificity and the two-sided exact 95% CoI. The index test was considered valid if the sum of sensitivity and specificity point estimates was significantly higher than 1, that is, if the odds ratio (OR) from logistic regression where the dependent variable was the recurrence status and the independent variable was the index test result (positive or negative) was >1. A minimum of 30 patients were targeted for enrollment for the present study, which was deemed sufficient to estimate sensitivity with 15% marginal error, assuming an ideal index test with 80% sensitivity and 80% specificity to RD and assuming 25% prevalence of RD [22]. All statistical analyses were reported according to best practices in clinical prediction models [23], and further details are presented in the Supplementary material.

### 2.6 Survival analyses

We correlated recurrence-free survival (RFS) with each preoperative score in the subset of patients with pathological diagnosis of M0 RCC from cohort 1 (Supplementary material). A univariable survival analysis was performed by fitting a Cox proportional hazard model to estimate the OR per unit of change of each preoperative score (0, 1, and 1 missing datum for plasma, urine, and combined, respectively) and the 95% CoI. In addition, two other variables were considered for the univariable Cox regression: age and Leibovich risk score. A multivariable Cox model was prespecified using variables reaching statistical significance in the univariable analysis. The sample size was not powered for this analysis given that survival was not intended as a primary endpoint.

## 3 Results

### 3.1 Patient characteristics

**Fig. 1:**
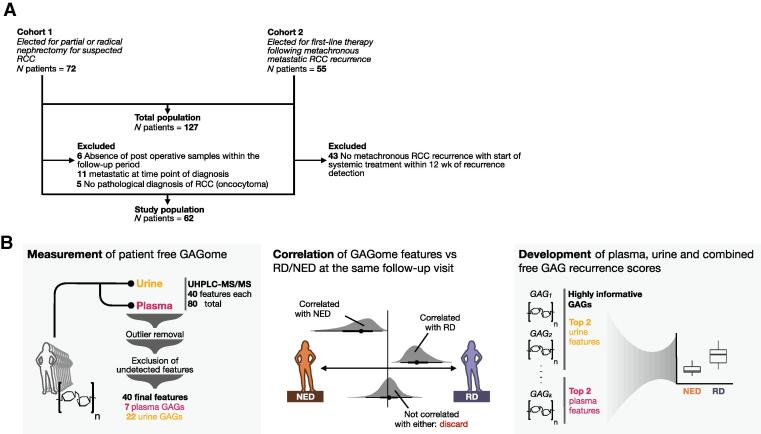
Study design: (A) patient flow and (B) workflow used to select free GAGome features correlated with recurrence and to develop GAGome-based recurrence scores. GAG = glycosaminoglycan; GAGome = glycosaminoglycan profile; NED = no evidence of disease; RCC = renal cell carcinoma; RD = recurrent disease; UHPLC-MS/MS = ultra-high-performance liquid chromatography and triple-quadrupole mass spectrometry.

The study design and patient flow are illustrated in Figure 1. We prospectively enrolled a total of 127 participants—72 were elected for partial or radical nephrectomy for suspected RCC (cohort 1) and 55 were scheduled for first-line therapy for metastatic RCC (cohort 2). Of the 127 patients in the total population, the study population included 50 patients with M0 RCC having undergone curative intent surgery from cohort 1 and 12 patients with metachronous RCC recurrence from cohort 2, for a total of 62 patients. The sample collection visits per patient and time to recurrence are shown in Supplementary Figure 1.

The median age was 63 year (interquartile range [IQR]: 56–70). The population was predominantly composed of males (65%, 40 males vs 22 females). The most common histological subtype was clear cell RCC (ccRCC; *N =* 51, 82%), followed by five papillary RCC (two type 1, two type 2, and one unspecified) and six chromophobe RCC cases. We listed other histopathological characteristics per cohort and across the study population in Table 1.

**Table 1:** Patients’ characteristics^a^

Factors	Total population			Study population		
	*N* = 127			*N* = 62		
	Cohort 1 (*N* = 72)	Cohort 2 (*N* = 55)	Both (*N* = 127)	Cohort 1 (*N* = 50)	Cohort 2 (*N* = 12)	Both (*N* = 62)
Age	64 (56–72)	69 (61–73)	66 (58–73)	63 (55–70)	66 (61–72)	63 (56–70)
Sex						
Female	21	18	39 (31)	19	3	22 (35)
Male	51	37	88 (69)	31	9	40 (65)
Histopathological diagnosis						
Oncocytoma	5	0	5 (4)	0	0	0
RCC	67	55	122 (96)	50	12	62 (100)
Clear cell	55	51	106 (84)	40	11	51 (82)
Papillary—type 1	4	0	4 (3)	2	0	2 (3)
Papillary—type 2	1	3	4 (3)	1	1	2 (3)
Papillary—unspecified	1	0	1 (1)	1	0	1 (2)
Chromophobe	6	1	7 (5)	6	0	6 (10)
Tumor size (cm)	7 (4.5–9)	NA	7 (4.5–9)	6 (4–9)	NA	6 (4–9)
TNM stage						
Stage I	28	0	28 (22)	23	0	23 (37)
Stage II	9	0	9 (7)	9	0	9 (15)
Stage III	18	0	18 (15)	17	0	17 (27)
Stage IV	12	0	12 (9)	1	0	1 (2)
Not available/not applicable	5	55	60 (47)	0	12	12 (19)
Fuhrman nuclear grade						
1	3	2	5 (4)	2	1	3 (5)
2	22	7	29 (23)	18	0	18 (29)
3	22	25	47 (37)	17	5	22 (35)
4	12	11	23 (18)	5	5	10 (16)
Not available/not applicable	13	10	20 (18)	8	1	9 (15)
Leibovich risk category						
Low	23	0	23 (18)	19	0	19 (31)
Intermediate	17	0	17 (13)	12	0	12 (19)
High	19	0	19 (15)	11	0	11 (18)
Not evaluated/not applicable	13	55	68 (54)	8	12	20 (32)
No. of visits with samples						
Preoperative	67	–	67	–	–	–
Postoperative follow-up	75	–	75	60	–	60
At systemic therapy start	–	55	55	–	12	12
No. of visits with investigator-assessed recurrence status						
Recurrent disease	–	–	–	5	12	17
No evidence of disease	–	–	–	55	0	55

NA = not applicable; RCC = renal cell carcinoma; TNM = tumor, node, metastasis.

a Data are presented as *n* (%) or as median (interquartile range).

In the total population (*N* = 127), there were 122 RCC (106 ccRCC and 16 non-ccRCC) and five oncocytoma patients. We listed other population characteristics per cohort and across cohorts in the total population in Table 1.

No adverse events were recorded during sample collection or recurrence status evaluation.

Overall, we measured the free GAGome in a total of 378 (195 plasma and 183 urine) samples in a single blinded central laboratory using a standardized UHPLC-MS/MS kit. No samples were identified as outliers.

### 3.2 Correlation between free GAGomes and radiological recurrence

In the study population (*N* = 62), we explored the correlation between free GAGomes and recurrence status using samples obtained at the same postoperative visit. The median follow-up time from curative intent surgery were 3 months (IQR: 1.8–7.8 months, maximum: 55 months). Each patient contributed on average 1.2 visits for a total of 72 visits. Throughout these, we recorded 17 (24%) RD and 55 (76%) NED cases. Plasma and urine samples were obtained, respectively, in 71 (99%) and 67 (93%) visits.

**Fig. 2:**
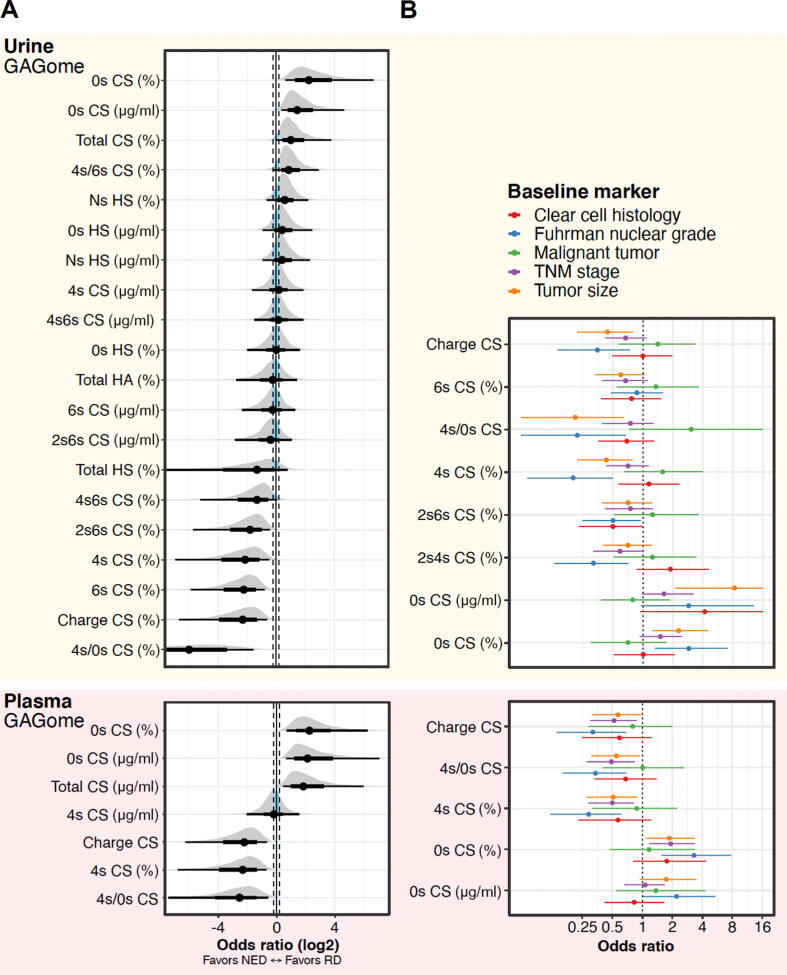
(A) Correlation of detectable plasma and urine free GAGome features in 72 postoperative visits with RD (*N* = 17) versus NED (*N* = 55) from 62 patients in the study population. The posterior probability density of the log-odds ratio for RD per unit of change of the free GAGome feature (in standard deviations from the mean value) is plotted together with the mean log-odds ratio and the 95% credible interval (CI; thick black line). The region of practical equivalence (ROPE) is marked by the two vertical dashed lines. A free GAGome feature is deemed compatible with RD or NED if its 95% CI does not fall inside the ROPE by >5%. (B) Correlation between plasma and urine free GAGome features compatible with RD and histopathological variables from 67 patients in the total population with preoperative samples in terms of log-odds ratio and 95% CI (horizontal line). CS = chondroitin sulfate; GAGome = glycosaminoglycan profile; HS = heparan sulfate; NED = no evidence of disease; RD = recurrent disease; TNM = tumor, node, metastasis.

We found that seven of 40 plasma and 20 of 40 urine free GAGome features were detectable (ie, measurable in the majority of samples). Of these, we determined that six (86%) of seven detectable features in plasma and eight (40%) of 20 detectable features in urine were compatible with RD versus NED (Fig. 2A, Supplementary Fig. 2 and 3, and Supplementary Table 1). In general, RD tended to correlate with plasma and urine free GAGomes featuring a higher concentration of unsulfated CS (0S CS) in both absolute and relative levels, and conversely a relatively lower concentration of certain monosulfated CS (4S CS in plasma and 6S CS in urine). Overall, we observed that recurrence was compatible with alterations in 14 (52%) of detectable free GAGome features.

### 3.3 Correlation of preoperative free GAGome features with histopathological variables

In the subset of the total population with preoperative samples from cohort 1 (*N* = 67), we observed that 11 (79%) of 14 preoperative free GAGome features compatible with recurrence were also compatible with at least one histopathological variable and in the same direction of correlation (Fig. 2B and Supplementary Table 2). In both plasma and urine, we observed compatible correlations with tumor size, Fuhrman nuclear grade, and TNM stage. We observed nominal correlations with malignant histology and clear cell histology, but these analyses appeared underpowered (only five benign and ten non–clear cell cases, respectively). Most of these correlations were with free GAGome features measured in relative concentrations (%). It was worth noting, however, that the absolute concentration of unsulfated CS in the urine was strongly compatible with large tumor size (>7 cm).

### 3.4 Development of plasma, urine, and combined free CS RCC recurrence scores

We developed three BART models to regress RD versus NED based on plasma or urine or combined free GAGome features. We manually curated the selection of two features for each model: for plasma, unsulfated CS (in μg/ml) and 4-sulfated CS (in %); and for urine, unsulfated CS (in μg/ml) and 6-sulfated CS (in %). For the combined model, we used as inputs the outputs of the plasma and urine models. The predicted probability of each model was scaled between 0 and 100, and henceforth referred to as plasma, urine, or combined free CS RCC recurrence score, given that the models rely solely on free CS. For example, if the plasma free CS RCC recurrence score is >50, then the probability of RD is >50% according to the model that uses only plasma features as predictors.

**Fig. 3:**
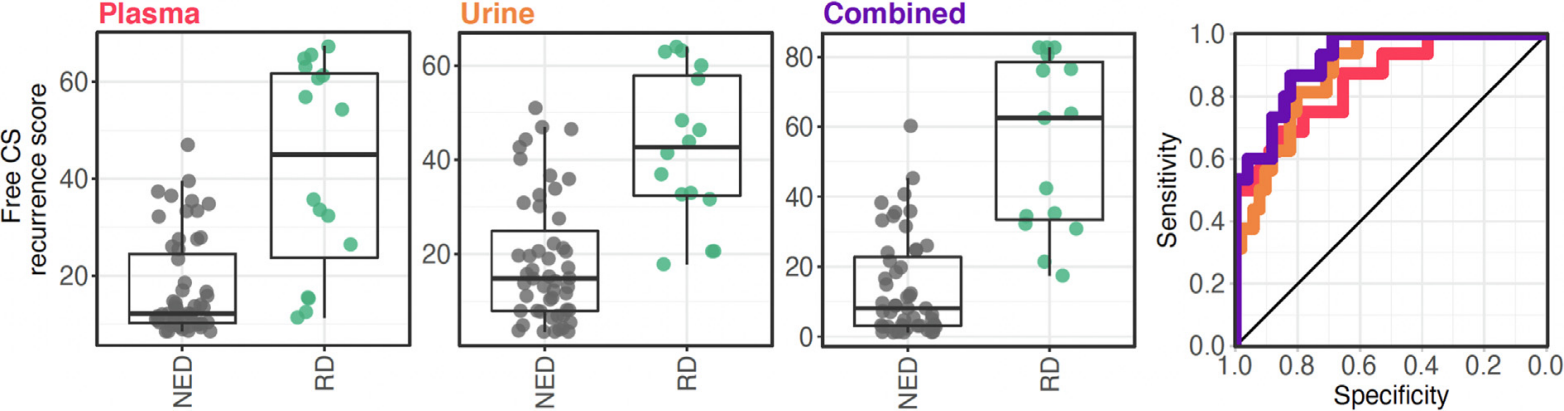
Plasma, urine, and combined free CS RCC recurrence scores in the study population (M0 RCC at postoperative visits) and their corresponding receiver operating characteristic curves (*N* = 62 patients across 72 visits: 16 RD vs 55 NED in 61 patients for plasma, 16 RD vs 51 NED for urine in 58 patients for urine, and 15 RD vs 51 NED in 57 patients for combined). CS = chondroitin sulfate; NED = no evidence of disease; RCC = renal cell carcinoma; RD = recurrent disease.

We evaluated the overall predictive performance of each score by internal validation using bootstrap resampling. For the plasma, urine, and combined free CS RCC recurrence scores, the scaled Brier scores were 0.42 (95% CoI: 0.23–0.62), 0.43 (95% CoI: 0.27–0.59), and 0.43 (95% CoI: 0.25–0.60) and the *R*^2^ values were 0.59 (95% CoI: 0.42–0.78), 0.64 (95% CoI: 0.49–0.81), and 0.64 (95% CoI: 0.48–0.81), respectively (Supplementary Fig. 4). In terms of discrimination between RD and NED, the AUCs were 0.91 (95% CoI: 0.85–0.97), 0.93 (95% CoI: 0.89–0.98), and 0.94 (95% CoI: 0.89–0.98) for the plasma, urine, and combined free CS RCC recurrence scores, respectively (Fig. 3). The calibration χ^2^ values were 2.6, 3.2, and 2.0 for the plasma, urine, and combined free CS RCC recurrence scores, respectively (Hosmer-Lemeshow test, *p* = 0.45, 0.35, and 0.58, respectively; Table 2).

**Table 2:** Diagnostic performance metrics for the classification of RD versus NED using plasma, urine, or combined free CS RCC recurrence scores at a postoperative visit (*N* = 62 patients across 72 visits: 16 RD vs 55 NED across 71 visits in 61 patients for plasma; 16 RD vs 51 NED across 67 visits in 58 patients for urine; 15 RD vs 51 NED across 66 visits in 57 patients for combined)^a^

Metric	Plasma	Urine	Combined
*Overall performance*			
Brier (scaled)	0.42 (0.23–0.62)	0.43 (0.27–0.59)	0.43 (0.25–0.60)
Nagelkerke R^2^	0.59 (0.42–0.78)	0.64 (0.49–0.81)	0.64 (0.48–0.81)
*Discrimination*			
AUC	0.91 (0.85–0.97)	0.93 (0.89–0.98)	0.94 (0.89–0.98)
*Calibration*			
Hosmer-Lemeshow test	χ^2^ = 2.6 (*p* = 0.45)	χ^2^ = 3.2 (*p* = 0.35)	χ^2^ = 2.0 (*p* = 0.58)
*Clinical usefulness*			
Cutoff	Predicted probability >20%		
Net benefit (%)	11.60	16	15.90
Interventions avoided (%)	33.80	44.80	50
Sensitivity	0.75 (0.48–0.93)	0.94 (0.70–1.00)	0.93 (0.68–1.00)
Specificity	0.73 (0.59–0.84)	0.67 (0.52–0.79)	0.73 (0.58–0.84)
Cutoff	Predicted probability >30%		
Net benefit (%)	9.80	12.70	12.10
Interventions avoided (%)	49.10	51.20	53.50
True positives	11	13	12
True negative	46	41	42
False positives	9	10	9
False negatives	5	3	3
Sensitivity	0.69 (0.41–0.89)	0.81 (0.54–0.96)	0.80 (0.52–0.96)
Specificity	0.84 (0.71–0.92)	0.80 (0.67–0.90)	0.82 (0.69–0.92)
Cutoff	Predicted probability >40%		
Net benefit (%)	10.30	7.50	10.60
Interventions avoided (%)	59.20	51.50	59.10
Sensitivity	0.50 (0.25–0.75)	0.56 (0.30–0.80)	0.60 (0.32–0.84)
Specificity	0.98 (0.90–1.00)	0.88 (0.76–0.96)	0.94 (0.84–0.99)

AUC = area under the receiving operating characteristic curve; CS = chondroitin sulfate; NED = no evidence of disease; RCC = renal cell carcinoma; RD = recurrent disease.

a The cutoffs used to determine RD was equivalent to the predicted probability of RD (in %). The 95% confidence interval values are given in brackets.

### 3.5 Clinical usefulness of plasma, urine, and combined free CS RCC recurrence scores

We used a decision curve analysis to determine the net benefit of using each free CS RCC recurrence score to inform the diagnosis of RD at different cutoff values (Supplementary Fig. 5). We defined a single cutoff of >30 for all scores, indicating that a predicted probability of RD of >30% would trigger a radiological evaluation. In other words, the clinician would be willing to undergo about two unnecessary radiological evaluations to diagnose one case of RD. At this cutoff, the sensitivity and specificity to RD were 69% and 84% in plasma (OR = 1.7, *p* < 0.001), 81% and 80% in urine (OR = 2.0, *p* < 0.001), and 80% and 82% when combined (OR = 2.1, *p* < 0.001), respectively (Table 2).

We examined the net benefit in two scenarios. In the first scenario, we imagined a follow-up visit in which there is no radiological evaluation of recurrence planned (“intervention in none” scenario). For example, the European Association of Urology (EAU) guidelines recommend that radiological evaluations are curtailed after 3 year of postoperative follow-up with NED in those with a low risk of recurrence [2]. In this scenario, the net benefit was 10%, 13%, and 12% for the plasma, urine, and combined free CS RCC recurrence scores, respectively (Table 2). As opposed to the strategy of performing no radiological evaluations in none of the patients, we estimated that performing the radiological evaluation based on a positive score was equivalent to a strategy that found ten, 13, and 12 cases of RD per hundred patients without any unnecessary imaging in plasma, urine, or combined scores, respectively.

In the second scenario, we imagined a follow-up visit in which there is a radiological evaluation of recurrence planned (“intervention in all” scenario). For example, the National Comprehensive Cancer Network guidelines recommend a radiological evaluation after 9 and 15 months after surgery as opposed to the EAU guidelines, indicating different willingness to intervene [24]. In this scenario, the interventions avoided were 49%, 51%, and 54% for the plasma, urine, and combined free CS RCC recurrence scores, respectively (Table 2). As opposed to the strategy of performing radiological evaluations in all patients, we estimated that performing imaging based on a positive score is equivalent to a strategy that avoids 49, 51, and 54 radiological evaluations per hundred patients without missing any RD in plasma, urine, or combined score, respectively.

In Table 2, we reported the clinical usefulness metrics also at a “low” cutoff (20%), which was more sensitive to RD and more acceptable in the first scenario when no radiological evaluations are routinely performed, and a “high” cutoff (40%), which was more specific to RD and more acceptable in the second scenario when radiological evaluations are performed in excess.

### 3.6 RFS and preoperative free CS RCC recurrence scores

In the subset of the total population with preoperative samples from cohort 1 and pathological diagnosis of M0 RCC (*N* = 51), there were seven (14%) recurrences at a median follow-up time of 11 months. In the univariable Cox regression analysis (Table 3), we observed a positive correlation between RFS and the preoperative combined free CS RCC recurrence score (hazard ratio [HR] = 1.90 [95% CoI: 1.10–3.29], *p* = 0.02), but only nominal correlations in plasma (HR = 1.38 [95% CoI: 0.92–2.09], *p* = 0.12) and urine (HR = 1.82 [95% CoI: 0.97–3.44], *p* = 0.06). In multivariable Cox regression adjusted for Leibovich risk points, the preoperative combined free CS RCC recurrence score was nominally correlated with RFS without reaching statistical significance (HR = 1.26 [95% CoI: 0.93–1.71], *p* = 0.14).

**Table 3:** Hazard ratio (HR) for RFS in univariable and multivariable Cox regression on each preoperative free CS RCC recurrence scores and other prognostic factors (patients with missing data were omitted)

Factors	*N* (*n* RD)	Univariable			Multivariable		
		HR	95% CI	*p* value	HR	95% CI	*p* value
Age	51 (7)	1.39	0.61–3.16	0.43			
Leibovich risk points	45 (7)	3.45	1.26–9.46	0.02	7.09	1.14–44.3	0.04
Plasma free CS RCC recurrence score	51 (7)	1.38	0.92–2.09	0.12			
Urine free CS RCC recurrence score	50 (7)	1.82	0.97–3.44	0.06			
Combined free CS RCC recurrence score	50 (7)	1.90	1.1–3.29	0.02	1.26	0.93–1.71	0.14

CI = credible interval; CS = chondroitin sulfate; RCC = renal cell carcinoma; RD = recurrent disease; RFS = recurrence-free survival.

## 4 Discussion

In this single-center prospective study, we discovered that plasma and urine free GAGomes changed in association with RCC recurrence after surgery. Although previous work demonstrated that GAGomes were altered in RCC compared with normal healthy levels [15], [16], their behavior at recurrence had not been investigated. The specific free GAGome features that altered with recurrence were overall consistent with changes previously associated with RCC compared with healthy controls both in urine [16] and in plasma [15], except for some inconsistencies in plasma sulfation patterns that we attribute to the fact that the present study focused on free rather than total GAGomes as in the previous study and total GAGomes include protein-bound GAGs.

We developed recurrence scores that incorporated the largest changes in plasma and/or urine free GAGomes. These included only CS features, such as an increase in unsulfated CS. This is consistent with a previously reported in vivo experiment using a syngeneic mouse model of RCC progression that caused the same increase in both plasma and urine [25]. Consistently, here unsulfated CS correlated with tumor size and grade. This suggests that the recurrence scores track a free GAGome feature that may causally be implicated with RCC. However, other factors may cause the here-observed aberrations in plasma and urine CS that may be specific or less to RCC, like inflammation-associated proteoglycans [26], glycocalyx degradation [27], [28], or renal and hepatic catabolism [29]. It will be important to elucidate the role of these factors to both improve the performance of the scores and understand potential sources of false positives and negatives.

Despite intense research on liquid biomarkers, none of these are recommended in the follow-up of RCC owing to the fact that virtually none of these reported prospective evidence of diagnostic performance in this setting [8]. In one of such studies, Wan et al [30] investigated prospectively total plasma cfDNA levels for monitoring postoperative recurrence events, reporting promising sensitivity and specificity to RD. However, the cutoff value and other critical performance parameters were not reported, rendering the interpretation of results for clinical practice difficult. In this context, our study represents one of the first prospective evaluations of liquid biomarkers for surveillance for RCC recurrence.

The free CS RCC recurrence scores appeared clinically useful. At different cutoffs, the free CS RCC recurrence scores, which are equivalent to the predicted probability of recurrence, had substantial net benefit compared with the strategy of performing imaging in all or no patients. This suggests that recurrence scores can considerably improve follow-up protocols of RCC patients, which are currently imaging intense with weak clinical benefits [6]. For example, we showed that performing a radiological evaluation based on a positive score could detect more recurrences or avoid unnecessary imaging with no extra harm for the patients. However, we also note that at present the sensitivity, which determines the false negative rate, appears insufficient to completely forego routine radiological evaluations. Beyond scientific evidence, the integration of free CS RCC recurrence scores in the clinical routine would also require determining the cost effectiveness of this extra procedure and benefits for patients and clinicians. We speculate that intercepting a patient when the recurrence is still local or oligometastatic may not only extend the number of quality-adjusted life years, for example, by opening a window for salvage treatments, but may also decrease the need of pricey systemic therapies indicated for metastatic RCC. The addition of free CS RCC recurrence scores to current follow-up protocols for surveillance for recurrence may therefore have positive health economics with substantial benefits for patients.

Limitations of this study include the relatively small event size (ie, number of recurrences) and a lack of external cohorts for validation. These two factors compound the risk of selection and detection biases. We believe that the prospective consecutive series cohort design of the study and the double-blind analysis of biomarker versus radiological results properly controlled for these biases. However, other factors such as inclusion of different RCC subtypes and median follow-up of <1 year may confound and limit the generalizability of the findings. The small sample size for these subsets cannot rule out that free GAGomes may not be as significantly altered in conjunction with long-term recurrences or in non–clear cell subtypes. Therefore, the generalizability of the here-reported performance of free CS RCC recurrence scores in a clinical setting should wait until evidence of external validation is produced in a more narrowly defined and larger M0 RCC population. This is, for example, investigated in the clinical study AURORAX-0087A (NCT04006405).

## 5 Conclusions

We conclude that plasma and urine free GAGomes were altered in association with RCC recurrence and that free CS RCC recurrence scores could substantially improve the follow-up of RCC patients after surgery. External validation of the scores is warranted prior to a wider implementation in the clinical routine.

  ***Author contributions*:** Jens Nielsen, Francesco Gatto and Sven Lundstam had collectively full access to all the data in the study and takes responsibility for the integrity of the data and the accuracy of the data analysis.

*Study concept and design*: Gatto, Stierner, Lundstam, Nielsen.

*Acquisition of data*: Lundstam, Stierner, Volpi, Maccari, Galeotti.

*Analysis and interpretation of data*: Gatto, Bratulic, Limeta.

*Drafting of the manuscript*: Gatto.

*Critical revision of the manuscript for important intellectual content*: All authors.

*Statistical analysis*: Gatto, Bratulic.

*Obtaining funding*: Gatto, Nielsen.

*Administrative, technical, or material support*: None.

*Supervision*: Gatto, Stierner, Lundstam, Nielsen.

*Other*: None.

  ***Financial disclosures:*** Jens Nielsen, Francesco Gatto and Sven Lundstam certify that all conflicts of interest, including specific financial interests and relationships and affiliations relevant to the subject matter or materials discussed in the manuscript (eg, employment/affiliation, grants or funding, consultancies, honoraria, stock ownership or options, expert testimony, royalties, or patents filed, received, or pending), are the following: At the study start, Francesco Gatto and Jens Nielsen were listed as coinventors in patent applications related to the biomarkers described in this study. At the time of submission, Francesco Gatto and Jens Nielsen were shareholders in Elypta AB, which owned the abovementioned patent applications. Francesco Gatto was an employee in Elypta AB, and Jens Nielsen was a board director. Saeed Dabestani received advisory fees from Elypta AB. All other authors declare no relevant conflicts of interest.

  ***Funding/Support and role of the sponsor*:** This work was financially supported by the Knut and Alice Wallenberg Foundation and Cancerfonden to Chalmers University of Technology (Jens Nielsen), and by VINNOVA (#2016-00763) and Västra Götaland Region to Chalmers University of Technology (Francesco Gatto).

## References

[1] Ferlay J., Colombet M., Soerjomataram I. (2021). Cancer statistics for the year 2020: an overview. Int J Cancer.

[2] EAU Guidelines Office EAU guidelines: renal cell carcinoma. Uroweb. https://uroweb.org/guideline/renal-cell-carcinoma/#1_4.

[3] Sun M., Choueiri T.K. (2016). Recurrence in renal cell carcinoma: the work is not done. Nat Rev Urol.

[4] Dabestani S., Beisland C., Stewart G.D. (2019). Long-term outcomes of follow-up for initially localised clear cell renal cell carcinoma: RECUR database analysis. Eur Urol Focus.

[5] Beisland C., Guðbrandsdottir G., Reisæter L.A.R., Bostad L., Hjelle K.M. (2016). A prospective risk-stratified follow-up programme for radically treated renal cell carcinoma patients: evaluation after eight years of clinical use. World J Urol.

[6] Dabestani S., Beisland C., Stewart G.D. (2019). Intensive imaging-based follow-up of surgically treated localised renal cell carcinoma does not improve post-recurrence survival: results from a European multicentre database (RECUR). Eur Urol.

[7] Jamil M.L., Keeley J., Sood A. (2020). Long-term risk of recurrence in surgically treated renal cell carcinoma: a post hoc analysis of the Eastern Cooperative Oncology Group-American College of Radiology Imaging Network E2805 trial cohort. Eur Urol.

[8] Campi R., Stewart G.D., Staehler M. (2021). Novel liquid biomarkers and innovative imaging for kidney cancer diagnosis: what can be implemented in our practice today? A systematic review of the literature. Eur Urol Oncol.

[9] Bratulic S., Gatto F., Nielsen J. (2021). The translational status of cancer liquid biopsies. Regen Eng Transl Med.

[10] Lasseter K., Nassar A.H., Hamieh L. (2020). Plasma cell-free DNA variant analysis compared with methylated DNA analysis in renal cell carcinoma. Genet Med.

[11] Nuzzo P.V., Berchuck J.E., Korthauer K. (2020). Detection of renal cell carcinoma using plasma and urine cell-free DNA methylomes. Nat Med.

[12] Li H., Bullock K., Gurjao C. (2019). Metabolomic adaptations and correlates of survival to immune checkpoint blockade. Nat Commun.

[13] Afratis N., Gialeli C., Nikitovic D. (2012). Glycosaminoglycans: key players in cancer cell biology and treatment. FEBS J.

[14] Karamanos N.K. (2019). Extracellular matrix: key structural and functional meshwork in health and disease. FEBS J.

[15] Gatto F., Blum K.A., Hosseini S.S. (2018). Plasma glycosaminoglycans as diagnostic and prognostic biomarkers in surgically treated renal cell carcinoma. Eur Urol Oncol.

[16] Gatto F., Volpi N., Nilsson H. (2016). Glycosaminoglycan profiling in patients’ plasma and urine predicts the occurrence of metastatic clear cell renal cell carcinoma. Cell Rep.

[17] Gatto F., Maruzzo M., Magro C., Basso U., Nielsen J. (2016). Prognostic value of plasma and urine glycosaminoglycan scores in clear cell renal cell carcinoma. Front Oncol.

[18] Tamburro D., Bratulic S., Abou Shameh S. (2021). Analytical performance of a standardized kit for mass spectrometry-based measurements of human glycosaminoglycans. J Chromatogr B.

[19] Bratulic S., Limeta A., Maccari F. (2022). Analysis of normal levels of free glycosaminoglycans in urine and plasma in adults. J Biol Chem.

[20] Chipman H.A., George E.I., McCulloch R.E. (2010). BART: Bayesian Additive Regression Trees. Ann Appl Stat.

[21] Vickers A.J., van Calster B., Steyerberg E.W. (2019). A simple, step-by-step guide to interpreting decision curve analysis. Diagn Progn Res.

[22] Hajian-Tilaki K. (2014). Sample size estimation in diagnostic test studies of biomedical informatics. J Biomed Inform.

[23] Steyerberg E.W., Vickers A.J., Cook N.R. (2010). Assessing the performance of prediction models: a framework for traditional and novel measures. Epidemiology.

[24] National Comprehensive Cancer Network. Clinical practice guidelines in oncology (NCCN Guidelines®)—Kidney cancer, v.2.2016. National Comprehensive Cancer Network Website. https://www2.tri-kobe.org/nccn/guideline/archive/urological2016-2017/english/kidney.pdf. 10.6004/jnccn.2004.0021 19795607

[25] Gatto F., Bratulic S., Cavarretta I.T.R. (2021). Detection of any-stage cancer using plasma and urine glycosaminoglycans. J Clin Oncol.

[26] Lord M.S., Day A.J., Youssef P. (2013). Sulfation of the bikunin chondroitin sulfate chain determines heavy chain hyaluronan complex formation. J Biol Chem.

[27] Koźma E.M., Kuźnik-Trocha K., Winsz-Szczotka K. (2020). Significant remodeling affects the circulating glycosaminoglycan profile in adult patients with both severe and mild forms of acute pancreatitis. J Clin Med.

[28] Schmidt E.P., Overdier K.H., Sun X. (2016). Urinary glycosaminoglycans predict outcomes in septic shock and acute respiratory distress syndrome. Am J Respir Crit Care Med.

[29] Pecly I.M.D., Melo-Filho N.M., Mourão P.A.S. (2006). Effects of molecular size and chemical structure on renal and hepatic removal of exogenously administered chondroitin sulfate in rats. Biochim Biophys Acta.

[30] Wan J., Zhu L., Jiang Z., Cheng K. (2013). Monitoring of plasma cell-free DNA in predicting postoperative recurrence of clear cell renal cell carcinoma. Urol Int.

