# Supplementary material for: Plasma and Urine Free Glycosaminoglycans as Monitoring Biomarkers in Nonmetastatic Renal Cell Carcinoma—A Prospective Cohort Study
Source: Eur Urol Open Sci. 2022 Jun 29;42:30–9. doi: 10.1016/j.euros.2022.06.003 (PMC9334826; doi:10.1016/j.euros.2022.06.003)
Supplement: Supplementary data 1 [file mmc1.docx]

**Supplementary material**

**Plasma and Urine Free Glycosaminoglycans as Biomarkers in Non-Metastatic Renal Cell Carcinoma – a Prospective Cohort Study**

**STARD checklist.**

|  | **Section & Topic** | **No** | **Item** | **Reported on page #** |
| --- | --- | --- | --- | --- |
|  |  |  |  |  |
|  | **TITLE OR ABSTRACT** |  |  |  |
|  |  | **1** | Identification as a study of diagnostic accuracy using at least one measure of accuracy  (such as sensitivity, specificity, predictive values, or AUC) | 2 |
|  | **ABSTRACT** |  |  |  |
|  |  | **2** | Structured summary of study design, methods, results, and conclusions  (for specific guidance, see STARD for Abstracts) | 2 |
|  | **INTRODUCTION** |  |  |  |
|  |  | **3** | Scientific and clinical background, including the intended use and clinical role of the index test | 3 |
|  |  | **4** | Study objectives and hypotheses | 3 |
|  | **METHODS** |  |  |  |
|  | *Study design* | **5** | Whether data collection was planned before the index test and reference standard  were performed (prospective study) or after (retrospective study) | 4 |
|  | *Participants* | **6** | Eligibility criteria | 4, Text S2 |
|  |  | **7** | On what basis potentially eligible participants were identified  (such as symptoms, results from previous tests, inclusion in registry) | 4, Text S2 |
|  |  | **8** | Where and when potentially eligible participants were identified (setting, location and dates) | 4 |
|  |  | **9** | Whether participants formed a consecutive, random or convenience series | 4 |
|  | *Test methods* | **10a** | Index test, in sufficient detail to allow replication | 4,5, Text S2 |
|  |  | **10b** | Reference standard, in sufficient detail to allow replication | 4, Text S2 |
|  |  | **11** | Rationale for choosing the reference standard (if alternatives exist) | 6, Text S2 |
|  |  | **12a** | Definition of and rationale for test positivity cut-offs or result categories  of the index test, distinguishing pre-specified from exploratory | 6, Text S2 |
|  |  | **12b** | Definition of and rationale for test positivity cut-offs or result categories  of the reference standard, distinguishing pre-specified from exploratory | 6, Text S2 |
|  |  | **13a** | Whether clinical information and reference standard results were available  to the performers/readers of the index test | 4, Text S2 |
|  |  | **13b** | Whether clinical information and index test results were available  to the assessors of the reference standard | 4, Text S2 |
|  | *Analysis* | **14** | Methods for estimating or comparing measures of diagnostic accuracy | 5, Text S2 |
|  |  | **15** | How indeterminate index test or reference standard results were handled | Text S2 |
|  |  | **16** | How missing data on the index test and reference standard were handled | Text S2 |
|  |  | **17** | Any analyses of variability in diagnostic accuracy, distinguishing pre-specified from exploratory | 5, Text S2 |
|  |  | **18** | Intended sample size and how it was determined | Text S2 |
|  | **RESULTS** |  |  |  |
|  | *Participants* | **19** | Flow of participants, using a diagram | Figure 1A, pg 9 |
|  |  | **20** | Baseline demographic and clinical characteristics of participants | Table 1, pg 7,8 |
|  |  | **21a** | Distribution of severity of disease in those with the target condition | Table 1, pg 7,8 |
|  |  | **21b** | Distribution of alternative diagnoses in those without the target condition | NA |
|  |  | **22** | Time interval and any clinical interventions between index test and reference standard | NA |
|  | *Test results* | **23** | Cross tabulation of the index test results (or their distribution)  by the results of the reference standard | Table 2, pg 15 |
|  |  | **24** | Estimates of diagnostic accuracy and their precision (such as 95% confidence intervals) | Table 2, pg 15 |
|  |  | **25** | Any adverse events from performing the index test or the reference standard | 6 |
|  | **DISCUSSION** |  |  |  |
|  |  | **26** | Study limitations, including sources of potential bias, statistical uncertainty, and generalisability | 17 |
|  |  | **27** | Implications for practice, including the intended use and clinical role of the index test | 17 |
|  | **OTHER INFORMATION** |  |  |  |
|  |  | **28** | Registration number and name of registry | 4 |
|  |  | **29** | Where the full study protocol can be accessed | NA |
|  |  | **30** | Sources of funding and other support; role of funders | 18 |
|  |  |  |  |  |

**Supplementary Methods.**

**Eligibility criteria.** Inclusion criteria for Cohort 1 were: elected for partial or radical nephrectomy for suspicion of RCC, Eastern Cooperative Oncology Group (ECOG) performance status 0-2; standard imaging evaluation within 12 weeks before inclusion; planned for standard imaging 3-6 months after surgery. Exclusion criteria for Cohort 1 were: multiple malignancies. Inclusion criteria for Cohort 2 were: elected for first-line drug therapy for metastatic RCC; standard imaging evaluation within 4 weeks before inclusion; ECOG performance status 0-2. Exclusion criteria for Cohort 2 were: prior antineoplastic systemic therapy; multiple malignancies. Eligible patients from Cohort 1 and 2 represented the total population.

We defined additional eligibility criteria to determine the population used for the primary analysis (study population). Inclusion criteria for Cohort 1: pathologic diagnosis of RCC; non-metastatic (M0) disease at time point of diagnosis. Exclusion criteria for Cohort 1: absence of post-operative samples within the follow-up period. Inclusion criteria for Cohort 2: metachronous RCC recurrence with start of systemic treatment within 12 weeks of recurrence detection. Exclusion criteria for Cohort 2: none.

**Assessment of recurrence.** Recurrence status was assessed based on clinical findings and radiological scans as read by experience uroradiologists and compatible with RCC local or distal recurrence. Final clinical determination of a recurrence was assessed by the treating physicians in a multidisciplinary team conference setting. The radiological definition of a recurrence was any detected lesion unequivocally deemed as a RCC metastasis or local recurrence. In case of uncertainty, a suspected metastatic lesion was biopsied for definitive determination of its RCC origin.

**GAGome measurements.** Laboratory measurements of the free GAGome included the absolute concentration in µg/mL of chondroitin sulfate (CS), heparan sulfate (HS), hyaluronic acid (HA) disaccharides, resulting in 17 independently measured features. Specifically, we quantified 8 CS disaccharides (0S CS, 2S CS, 6S CS, 4S CS, 2S6S CS, 2S4S CS, 4S6S CS, TriS CS) and 8 HS disaccharides (0S HS, 2S HS, 6S HS, NS HS, NS6S HS, NS2S HS, 2S6S HS, TriS HS) – corresponding to different sulfation patterns of CS and HS – and 1 HA disaccharide. The GAGome was expanded to include an additional 23 dependent features for a total of 40.

**Correlation analysis of free GAGome features and recurrence status.** The analysis was performed in the study population. A patient could contribute more than one visit. We regressed recurrence status as a binary outcome on each detectable free GAGome feature using a Bayesian mixed-effects logistic regression model that included a patient-specific intercept.

The posterior probability of recurrence (RD vs. NED) conditional to the value of a given free GAGome feature was interpreted as the log-odds ratio (OR) for RD per unit of change (in standard deviations) from the mean value of that free GAGome feature. We computed the OR 95% credible interval (95% CI) for each free GAGome feature from the corresponding posterior probability density. We regarded a free GAGome feature as compatible (i.e. correlated) with recurrence if the OR 95% CI did not cross 0 and no more than 5% fell inside the (-10%, +10%) interval around 0, referred to as the region of practical equivalence (ROPE).

**Correlation analysis between free GAGome features and histopathologic variables.** The analysis was performed in the total population. We correlated each pre-operative free GAGome feature with histopathologic variables in all patients elected for partial or radical nephrectomy for RCC from Cohort 1. We performed a univariable logistic or ordinal Bayesian regression between each free GAGome feature as the explanatory variable and each of the following histopathologic variables as the response variable: tumor size (<= 7 cm vs. > 7 cm, binary, no missing data, RCC patients only), Fuhrman nuclear grade (G1-2 vs. G3-4, binary, 1 missing datum, clear cell RCC patients only), TNM stage (stage I-II vs. stage III vs. stage IV, ordinal, no missing data, RCC patients only), malignant or benign lesion (RCC vs. non-RCC, binary, no missing data), RCC subtype (clear cell vs. non-clear cell, binary, no missing data and RCC patients only). A correlation was regarded compatible with the data if the 95% credibility interval for the log-odds-ratio estimate on the free GAGome feature did not include 1.

**Development of free CS RCC recurrence scores.** We developed free GAGome-based scores in the study population. For each fluid (plasma and urine), we manually ranked free GAGome features by compatibility with recurrence, correlation with histopathologic variables (or lack thereof), and a mixed use of relative and absolute concentrations. Two free GAGome features were used as inputs to train two Bayesian Additive Regression Trees (BART) models^1^ - one for plasma, one for urine. A third BART model was developed by using as inputs the outputs of the plasma and urine BART models. Each BART model was trained to classify recurrence as a binary outcome (RD versus NED) given the free GAGome features measured at the same post-operative visit in which recurrence status was determined. We normalized the output of each model in the range of 0 to 100 to define three free CS RCC recurrence scores - one for plasma, one for urine, and one combined – so that the score is equivalent to the predicted probability of RD (in %). The three free CS RCC recurrence scores were the index tests for this study. They were provided as described in the section “Data Availability”.

**Statistical analysis for the diagnostic performance of free CS RCC recurrence scores.** We assessed the predictive performance of each score by internal validation using bootstrap resampling (*N* = 500 bootstraps). We reported the predictive performance in terms of scaled Brier score (in %) and Nagelkerke *R^2^* (in %) with empirical 95% confidence intervals as determined across bootstraps.

We assessed the discrimination performance of the three free CS RCC recurrence scores (plasma, urine, combined) in terms of area-under-the-receiving operating characteristic-curve (AUC) for the classification of RD versus NED according to each score versus the observed recurrence status according to the reference standard. The reference standard, including its assessment as positive to RD, was described above, and chosen as representative of standard of care. The patient population was the study population as defined above. The results of each index test were not available to the investigator (as reference standard assessor) and vice versa. We assessed calibration visually by grouping the visits with RD in quartiles and plotting the resulting proportions against the predicted probability for RD in the same visit according to each free CS RCC recurrence score. The goodness-of-fit and corresponding c^2^ statistics was tested using the Hosmer-Lemeshow test.

We used decision curve analysis to determine a single test positivity cut-off for all free CS RCC recurrence scores so that the net benefit over the base scenarios “intervention for all” (i.e. radiological evaluation for all) and “intervention for none” (i.e. radiological evaluation for none) was maximized^2^. We determined the clinical usefulness of each score by cross-tabulating RD versus NED as predicted by the free CS RCC recurrence score versus the recurrence status as observed using the reference standard. We performed three distinct analyses, one per score (plasma, urine, and combined). There were no indeterminate free CS RCC recurrence scores or reference standard results. Missing evaluations of free CS RCC recurrence scores were excluded and assumed rare and random. There were no missing reference standard results. We used the three confusion matrices (one per score) resulting from this analysis to determine the specificity and sensitivity of the corresponding free CS RCC recurrence score to RD and their 95% confidence interval (CoI). No variability analyses of diagnostic accuracy were performed.

The index test was considered valid if the sum of sensitivity and specificity point estimates were significantly higher than 1, i.e. if the odds ratio (OR) from logistic regression where the dependent variable was the recurrence status and the independent variable was the index test result (positive or negative) was > 1. The precision of the sensitivity and specificity estimates was computed as the two-sided exact 95% confidence interval of the point estimate. Given that no prior estimates on the sensitivity and specificity were known for the index test at the study start, the sample size was powered targeting a minimum of 30 patients sufficient to estimate sensitivity with 15% marginal error assuming an ideal index test with 80% sensitivity and 80% specificity to RD and assuming 25% prevalence of RD.

**Survival analyses.** In the total population, we correlated recurrence-free survival (RFS) with each pre-operative score in the subset of patients with pathologic diagnosis of M0 RCC from Cohort 1. RFS was calculated as the time between the date of sampling and the time of event. The time of event is defined as right-censoring (date of last follow-up without the event) or as date of first radiological recurrence (defined above as RD). Univariable survival analysis was performed by fitting a Cox proportional hazard model to estimate the odds-ratio per unit of change of each pre-operative score (0, 1, 1 missing datum for plasma, urine, and combined, respectively) and the 95% confidence interval. In addition, two other variables were considered for regression of survival using a univariable Cox model: age (continuous, in years, no missing data), and Leibovich risk score (continuous, range 0-11, 4 missing data – note that this score was computed also for non-clear cell RCC since its prognostic performance was recently validated in these subtypes^3^). Missing data were omitted. A multivariable Cox model was pre-specified using variables reaching statistical significance in the univariable analysis. The validity of the proportional hazard assumption was checked using a two-sided t-test between transformed survival time and the scaled Schoenfeld residuals. The sample size was not powered for this analysis given that survival was not intended as a primary endpoint. We checked for overfitting by performing internal validation of the univariable and multivariable models using a bootstrapping algorithm (1,000 bootstraps) and observing the change in Somers’ *D* rank correlation (*D_xy_*) statistics in the original datasets as opposed to the test set. A change < 25% was considered acceptable.

Statistical analyses were performed using the packages *brms* (2.14.4), *BART* (2.9)*, epiR* (1.0.15)*, rms* (6.0.1)*, survival* (3.2.7), *tidybayes* (2.3.1), and *tidyversse* (1.3.0) in R programming language, v. 4.0.5. *p* values < 0.05 were considered significant.

**Data Availability:** The code used for the computation of free CS RCC recurrence scores can be requested to the authors.

**Supplementary Table 1.** Log-odds ratios for RD versus NED for each detectable urine and plasma free GAGome feature (in brackets, the 95% Credibility Interval (CI)). Free GAGome features were normalized by mean-centering and scaling by the standard deviation. Log-odds ratios compatible with a change in a free GAGome features outside the Region of Practical Equivalence (ROPE) by <5% are highlighted in bold. Key: CS – chondroitin sulfate; HS – heparan sulfate.

| **Free GAGome feature** | **Log-Odds Ratio [95% CI]** | **Percentage of CI** **in ROPE** |
| --- | --- | --- |
| **4S/0S CS urine ratio** | **-6.92 [-15.21, -0.49]** | **0** |
| **Charge CS urine** | **-2.66 [-5.88, -0.39]** | **0** |
| **0S CS urine [%]** | **2.61 [0.36, 5.73]** | **0** |
| **6S CS urine [%]** | **-2.53 [-5.22, -0.54]** | **0** |
| **2S6S CS urine [%]** | **-2.13 [-4.90, -0.17]** | **0.14** |
| **4S CS urine [%]** | **-2.52 [-5.76, -0.13]** | **0.22** |
| **0S CS urine [μg/mL]** | **1.71 [0.13, 4.01]** | **0.46** |
| **4S6S CS urine [%]** | **-1.63 [-4.35, 0.38]** | **4.61** |
| Total CS urine [μg/mL] | 1.22 [-0.22, 3.28] | 5.46 |
| 4S/6S CS urine ratio | 0.98 [-0.46, 2.69] | 8.57 |
| Total HS urine [μg/mL] | -1.96 [-6.99, 1.31] | 11.14 |
| NS HS urine [%] | 0.64 [-0.73, 2.10] | 15.58 |
| 0S HS urine [μg/mL] | 0.51 [-1.07, 2.31] | 22.01 |
| 2S6S CS urine [μg/mL] | -0.52 [-2.59, 1.23] | 22.41 |
| NS HS urine [μg/mL] | 0.48 [-1.05, 2.18] | 22.74 |
| Total HA urine [μg/mL] | -0.35 [-2.49, 1.64] | 23.43 |
| 6S CS urine [μg/mL] | -0.32 [-2.29, 1.35] | 24.44 |
| 4S CS urine [μg/mL] | 0.17 [-1.49, 1.99] | 26.65 |
| 4S6S CS urine [μg/mL] | 0.16 [-1.44, 1.90] | 26.71 |
| 0S HS urine [%] | -0.04 [-1.88, 1.66] | 26.81 |

| **4S/0S CS plasma ratio** | **-2.89 [-6.42, -0.23]** | **0** |
| --- | --- | --- |
| **4S CS plasma [%]** | **-2.69 [-5.81, -0.35]** | **0** |
| **0S CS plasma [μg/mL]** | **2.59 [0.34, 6.00]** | **0** |
| **0S CS plasma [%]** | **2.56 [0.37, 5.50]** | **0** |
| **Charge CS plasma** | **-2.54 [-5.46, -0.39]** | **0** |
| **Total CS plasma [μg/mL]** | **2.17 [0.15, 5.08]** | **0.15** |
| 4S CS plasma [μg/mL] | -0.23 [-1.98, 1.57] | 25.37 |

**Supplementary Table 2.** Correlation between each pre-operative free GAGome feature compatible with RD (N_features_ = 14) and different histopathological variables in univariable logistic or ordinal Bayesian regression (N_patients_ = 67, 2 missing urine). Compatible associations are highlighted in bold.

| **Free GAGome feature** | **Odds-ratio (95% credibility interval)** | | | | **Cumulative odds-ratio (95% credibility interval)** |
| --- | --- | --- | --- | --- | --- |
|  | **Tumor size (RCC only, < 7 cm (55%) vs. > 7 cm (45%), N = 62)** | **Fuhrman nuclear grade (ccRCC only, G1-2 (37%) vs G3-4 (63%), N = 51)** | **Subtype (RCC only, clear cell (84%) vs. not (16%), N = 62)** | **Malignant tumor (RCC (93%) vs. not (7%), N = 67)** | **TNM stage (RCC only, Stage I-II (55%) vs. Stage III (26%) vs. Stage IV (19%), N = 62)** |
| 0S CS plasma [%] | **1.87 (1.08 - 3.33)** | **3.3 (1.56 - 7.83)** | 1.76 (0.82 - 4.32) | 1.17 (0.47 - 3.33) | **1.93 (1.17 - 3.33)** |
| 0S CS plasma [μg/mL] | 1.74 (0.94 - 3.5) | **2.21 (1.02 - 5.39)** | 0.83 (0.42 - 1.66) | 1.37 (0.55 - 4.3) | 1.07 (0.66 - 1.67) |
| 4S CS plasma [%] | **0.51 (0.28 - 0.88)** | **0.29 (0.12 - 0.61)** | 0.57 (0.23 - 1.22) | 0.88 (0.32 - 2.22) | **0.5 (0.29 - 0.83)** |
| 4S/0S CS plasma | **0.55 (0.31 - 0.95)** | **0.34 (0.16 - 0.68)** | 0.68 (0.33 - 1.38) | 1.01 (0.4 - 2.61) | **0.49 (0.28 - 0.84)** |
| Charge CS plasma | **0.57 (0.32 - 0.97)** | **0.32 (0.14 - 0.68)** | 0.59 (0.25 - 1.24) | 0.8 (0.29 - 2) | **0.52 (0.3 - 0.87)** |
| Total CS plasma [μg/mL] | 1.11 (0.66 - 1.88) | 1.04 (0.57 - 1.88) | 0.43 (0.2 - 0.87) | 1.04 (0.43 - 2.77) | 0.63 (0.36 - 1.05) |
| 0S CS urine [%] | **2.29 (1.26 - 4.51)** | **2.89 (1.33 - 7.09)** | 1.01 (0.51 - 2.07) | 0.71 (0.3 - 1.73) | 1.5 (0.93 - 2.45) |
| 0S CS urine [μg/mL] | **8.3 (2.11 - 40.68)** | 2.88 (0.96 - 12.85) | 4.19 (0.95 - 31.55) | 0.79 (0.38 - 1.87) | 1.63 (0.99 - 3.2) |
| 2S6S CS urine [%] | 0.71 (0.39 - 1.24) | **0.5 (0.25 - 0.94)** | **0.5 (0.23 - 0.99)** | 1.25 (0.51 - 3.64) | 0.75 (0.42 - 1.26) |
| 4S CS urine [%] | **0.43 (0.22 - 0.78)** | **0.2 (0.07 - 0.5)** | 1.15 (0.57 - 2.31) | 1.58 (0.65 - 3.99) | 0.71 (0.43 - 1.15) |
| 4S/0S CS urine | **0.21 (0.06 - 0.64)** | **0.22 (0.06 - 0.67)** | 0.69 (0.36 - 1.3) | 3.06 (0.73 - 29.65) | 0.75 (0.39 - 1.27) |
| 4S6S CS urine [%] | 0.75 (0.43 - 1.28) | 0.87 (0.47 - 1.57) | 2 (0.93 - 4.7) | 0.97 (0.4 - 2.5) | 1.16 (0.69 - 1.95) |
| 6S CS urine [%] | 0.6 (0.33 - 1.04) | 0.87 (0.48 - 1.59) | 0.77 (0.38 - 1.53) | 1.35 (0.55 - 3.61) | 0.67 (0.39 - 1.11) |

**Supplementary Figure 1.** Swimmer plot of all patients in Cohort 1 (N = 50, top) and Cohort 2 (N = 12, bottom) illustrating the baseline visit, the follow-up visits, and the time of recurrence or end of follow-up from the date of surgery (in weeks). Visits in which only plasma was collected are shown with a pink contour.

**Supplementary Figure 2.** Plasma free GAGome features compatible with RD (*N* = 16) versus NED (*N* = 55) in 61 M0 RCC patients. Concentrations are in μg mL^-1^ (labelled [ug/mL]) or in mass fractions (mg mg_total_^-1^ in %).

**Supplementary Figure 3.** Urine free GAGome features compatible with RD (*N* = 16) versus NED (*N* = 51) in 58 M0 RCC patients. Concentrations are in μg mL^-1^ (labelled [ug/mL]) or in mass fractions (mg mg_total_^-1^ in %).


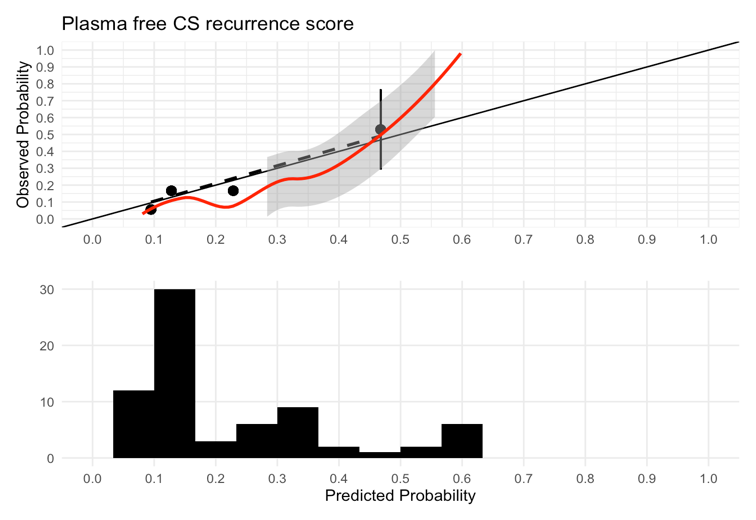


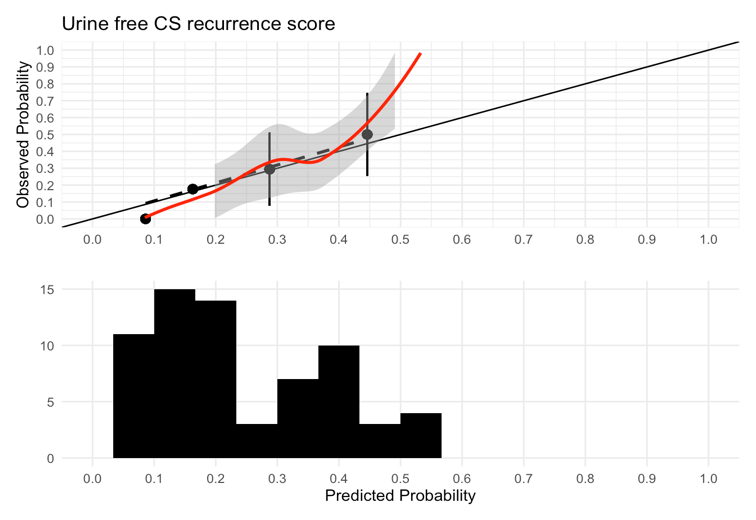


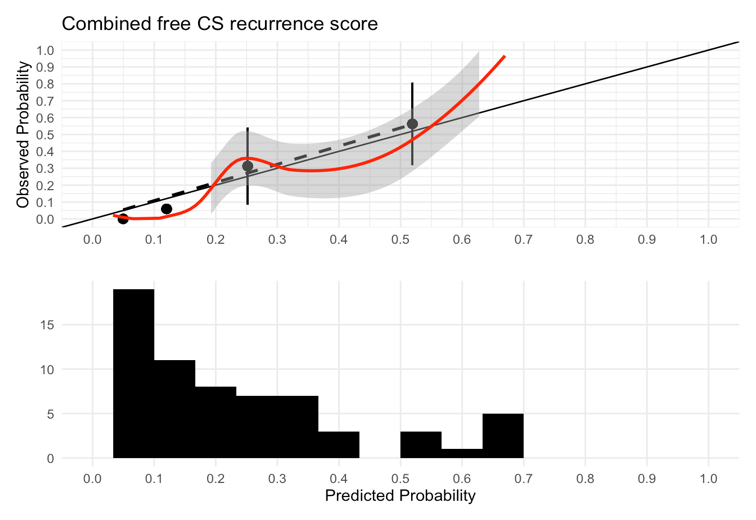


**Supplementary Figure 4.** Calibration curve for the predicted vs. observed probability of RD according to the plasma (top), urine (middle) and combined (bottom) free CS RCC recurrence scores (*N_visits_* = 71, 67, 66 respectively) and frequency at each 10%-predicted probability bin.


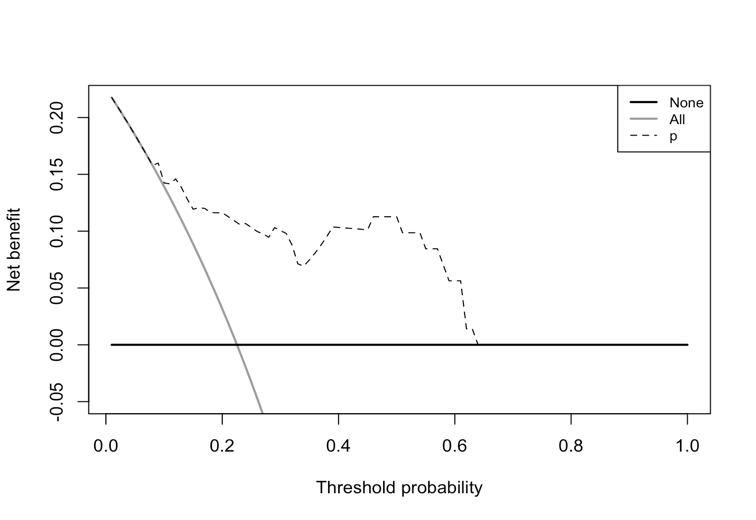


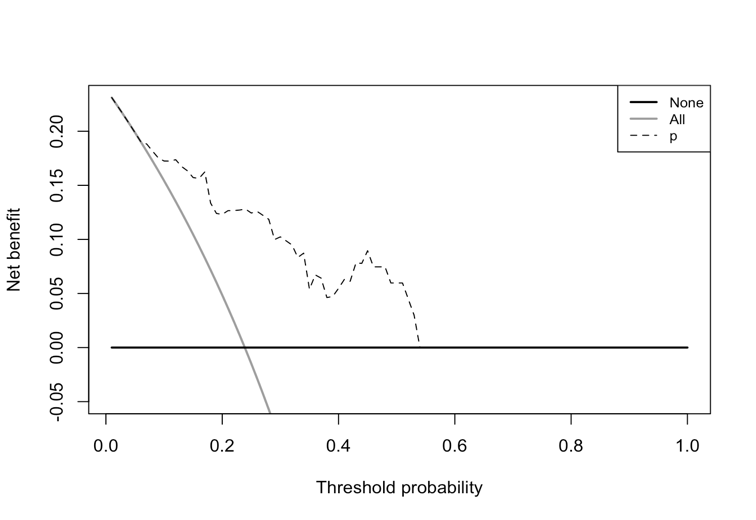


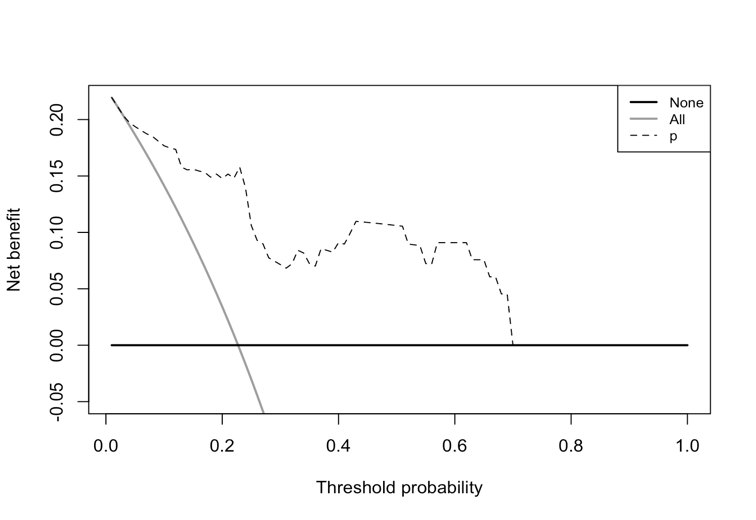


**Supplementary Figure 5.** Decision curve analysis on the net benefit of intervention (e.g. perform a CT scan for recurrence evaluation) based on a given predicted probability of RD cut-off for the plasma (top), urine (middle) and combined (bottom) free CS RCC recurrence scores (dashed line, ”p” - *N_visits_* = 71, 67, 66 respectively). In black and grey lines, the net benefit for the strategy to intervene in no patients (“None”) or in all visits (“All”), respectively.

**SUPPLEMENTARY REFERENCES**

1. Chipman HA, George EI, McCulloch RE. BART: Bayesian additive regression trees. *Ann Appl Stat*. 2010;4(1):266-298. doi:10.1214/09-AOAS285

2. Vickers AJ, van Calster B, Steyerberg EW. A simple, step-by-step guide to interpreting decision curve analysis. *Diagnostic and Prognostic Research*. 2019;3(1):18. doi:10.1186/s41512-019-0064-7

3. Oza B, Eisen T, Frangou E, et al. External Validation of the 2003 Leibovich Prognostic Score in Patients Randomly Assigned to SORCE, an International Phase III Trial of Adjuvant Sorafenib in Renal Cell Cancer. *JCO*. Published online February 25, 2022:JCO.21.01090. doi:10.1200/JCO.21.01090

4. Hajian-Tilaki K. Sample size estimation in diagnostic test studies of biomedical informatics. *J Biomed Inform*. 2014;48:193-204. doi:10.1016/j.jbi.2014.02.013
